# Oncology: Management of Elderly Cancer Patients

**DOI:** 10.1155/2018/7362585

**Published:** 2018-06-07

**Authors:** Meritxell Arenas, Nam Nguyen, Pierfrancesco Franco, Ugur Selek, Angeles Rovirosa, Sebastia' Sabater

**Affiliations:** ^1^Department of Radiation Oncology, Hospital Universitari Sant Joan de Reus, Tarragona, Spain; ^2^Department of Radiation Oncology, Howard University, Washington, DC, USA; ^3^Department of Oncology-Radiation Oncology, University of Turin, Turin, Italy; ^4^Department of Radiation Oncology, Koc University, Istanbul, Turkey; ^5^Department of Radiation Oncology, Hospital Clinic Universitari, Barcelona, Spain; ^6^Department of Radiation Oncology, Complejo Hospitalario de Albacete, Albacete, Spain

The aging of the general population in developed countries is becoming a major healthcare issue and it is commonly addressed as “Silver Tsunami.” Cancer is growing in burden in elderly patients, as almost 80% of the newly diagnosed patients are aged over 55 years and the median age at diagnosis for most neoplastic conditions is over 60 years [1, 2]. Adjunctively, tumor incidence for the population beyond 65 years has increased 11-fold in comparison to younger adults [3]. Elderly patients represent a peculiar setting for cancer therapy, with specific characteristics in terms of comorbid conditions, compliance to treatment, clinical endpoints, and psychological and social issues. Therefore, the optimal therapeutic strategy for elderly patients represents an intriguing clinical challenge. Most of the randomized clinical trials did not enroll older patients because of age limit and particular comorbid conditions as exclusion criteria [4]. In a recent survey performed by the European Organization for Research and Treatment of Cancer (EORTC) and dealing with health-related quality of life in elderly patients, authors observed that, among 6000 patients included in 25 trials, only 9% were aged ≥ 70 [5]. Hence the clinical evidence in this setting is lacking and most of the guidelines are relatively helpful. Another interesting aspect is the different perspective on treatment consequences between older and younger patient populations as, for example, younger patients seem to consider more worrisome the impact of cancer therapies on social role functioning and consequent financial issues, while elderly patients are more focused on items such as impaired physical functioning, appetite loss, and constipation [5]. Thus, older patients have specific needs and concerns. This setting of patients also poses several methodological issues for appropriate clinical management and future trial design. Compliance to treatment may be different than that of the general population and drug pharmacokinetics may undergo age-related modifications, calling for the need of tailored clinical strategies [4]. Hypofractionation schedules reduce acute toxicity, which can lead to discontinuation of radiotherapy treatment [6–8]. Appropriate screening tools for geriatric assessment and biomarkers for aging may strongly help during the clinical decision-making process [4]. Consolidated clinical endpoints, such as survival, may not perfectly fit the context and should be sustained by items related to quality of life and patient's reported outcomes. The oncological community is gaining awareness on this topic and several initiatives were set up to fulfil this gap. The Task Force for the Elderly within the EORTC organization produced a position paper on the treatment of elderly patients with cancer and developed a screening tool to distinguish “fit” elderly patients from fragile cases in order to correctly allocate them to the most proper treatment strategy [1, 9]. The American Society of Clinical Oncology (ASCO) formulated, within a statement paper, 5 recommendations to build up evidence-based guidelines to guide therapeutic strategy for elderly patients with cancer [10]. With the present special issue, we would like to contribute to the growing awareness in the treatment of cancer in older patients covering a broad range of fields of interest, including different therapeutic strategies (surgery, radiotherapy, chemotherapy, immunotherapy, and hormonal therapy), treatment tolerance and quality of life, clinical prognostic factors, biomarkers, and financial and social implications. The management of elderly patient with cancer is challenging and articulated, and the quest for the best option is yet ongoing. The right balance between cure and care should be the final goal. With this issue, we hope to help in raising the level of awareness and knowledge on this topic.

*Meritxell Arenas* *Nam Nguyen* *Pierfrancesco Franco* *Ugur Selek* *Angeles Rovirosa* *Sebastia' Sabater*
